# Effect Of Different Alkaline Treatments of Titanium Surface on Human Osteoblasts Metabolism

**DOI:** 10.1590/0103-6440202405786

**Published:** 2024-10-28

**Authors:** Talita Caira Silva, Lais M Cardoso, Taisa N Pansani, Edson Alfredo, Carlos de Souza-Costa, Fernanda Gonçalves Basso

**Affiliations:** 1 Department of Dentistry, Ribeirão Preto University(UNAERP), Ribeirão Preto, SP, Brazil; 2 São Paulo State University (UNESP) , School of Dentistry, Araraquara, SP, Brazil

**Keywords:** surface treatment, titanium, sodium hydroxide

## Abstract

This investigation demonstrates the effect of alkali modification of titanium on the metabolism of human osteoblasts. Polished titanium discs were subjected to alkalinization protocols with NaOH (5M) at 60°C or 120°C. Surface topography and roughness were evaluated using scanning electron microscopy (SEM). Osteoblasts were seeded onto titanium discs, followed by cell adhesion and viability analysis, total protein and collagen production, and alkaline phosphatase (ALP) activity. Gene expression of tumor necrosis factor-alpha (TNF-α) and beta-defensin 3 (HBD3) was evaluated after inflammatory stimulus with lipopolysaccharides (LPS) of *Porphyromonas gingivalis* (1 μg/mL) for 4 h. Discs subjected to modification with NaOH showed major irregularities, especially for 120°C-protocol. Increased adhered cell number was observed for surfaces modified by NaOH. Osteoblasts cultured on modified surfaces showed higher cell viability, total protein and collagen synthesis, and ALP activity than that of cells cultured on the polished discs. Osteoblast response to LPS exposure showed increased TNF-α gene expression by these cells when cultured on the polished discs, while increased expression of HBD3 was detected for all groups in the presence of LPS. Modification of titanium discs by NaOH at 60°C or 120°C promoted an increase in adhesion and metabolism of osteoblasts and favored the response to inflammatory stimulus.

## Resumo

Este estudo demonstra o efeito da modificação da superfície de titânio por alcalinização sobre o metabolismo de osteoblastos humanos. Discos de titânio polidos foram submetidos ao protocolo de alcalinização com NaOH (5M) a 60°C ou 120°C. A topografia e rugosidade superficial foram avaliados por microscopia eletrônica de varredura (MEV). Osteoblastos foram cultivados sobre discos de titânio, seguido de análise da adesão e viabilidade celular, síntese de proteína total e colágeno, além de atividade de fosfatase alcalina (ALP). A expressão genica do fator de necrose tumoral-alfa (TNF-α) e beta-defensina (HBD-3) foram avaliadas após estimulo inflamatório com lipopolissacarídeos (LPS) de *Porphyromonas gingivalis* (1 μg/mL) por 4 h. Os discos submetidos à modificação com NaOH demonstraram maiores irregularidades, especialmente para o protocolo de 120°C. Aumento da adesão celular foi observada para as superfícies modificadas por NaOH. Os osteoblastos cultivados sobre as superfícies modificadas apresentaram maior viabilidade, síntese de proteína total e de colágeno, além de maior atividade de ALP quando comparadas às células cultivadas sobre os discos polidos. Os osteoblastos apresentaram aumento da síntese de TNF-α em resposta ao LPS para as células cultivadas sobre os discos polidos, enquanto aumento da expressão de HBD-3 foi detectado para todos os grupos na presença de LPS. A modificação dos discos de titânio por NaOH a 60°C ou 120°C promoveu aumento da adesão e metabolismo de osteoblastos e favoreceu a resposta a estímulo inflamatório.

## Introduction

The osseointegration process consists of the intimate interaction between the implant and the alveolar bone, which is able to support masticatory forces. Bone tissue formation around implants is mediated mainly by osteogenic cells. This process involves several cellular and molecular events, starting with granulation tissue formation and activation of the hemostatic cascade, followed by an innate immune response, mediated by macrophages, aiming to eliminate possible pathogens and tissue debris. At the end of this inflammatory phase, the reparative phase begins, characterized by neovascularization and release of growth factors, such as vascular endothelial growth factor, and bone morphogenetic protein, among others, which stimulate the migration and differentiation of osteogenic cells. Such cells initiate the deposition of extracellular matrix, rich in type I collagen, followed by mineralization and remodeling ^1^.

Several factors can influence the osseointegration process, such as bone morphology and vascularity, condition of periodontal tissues, systemic conditions, and medications ^2^ ^)_^. In addition to patient-related factors, the type of material, topography, and chemical composition of implant surfaces can directly interfere with the process of bone neoformation and peri-implant maintenance ^3^.

Titanium is an excellent biomaterial as the gold standard for intraosseous implants when it comes to replacing dental elements. This material has shown good compatibility, compression resistance, and corrosion resistance ^4^.

Increasing the surface roughness of dental implants favors peri-implant tissue maintenance ^3^ ^)_^, through increased wettability, cell adhesion, and osseointegration, by increasing the contact angle of the material with the recipient tissue and allowing greater mechanical embrication with the bone matrix ^5^ ^)_^. Thus, the constant search for conditions that favor bone neoformation around implants has stimulated the development of different surface treatments for titanium dental implants in order to improve the adhesion, spreading, and differentiation of osteoblasts at the implant interface ^5^.

Several surface modifications have been proposed and evaluated, such as the addition of hydroxyapatite crystals and another blasting, high-intensity laser treatments, acid treatments, and alkaline treatments ^5^ ^,^ ^6^ ^)_^. Among these options, the alkaline treatment is an interesting alternative, because besides promoting a change in surface topography and wettability, this modification also favors the interaction of the surface with organic molecules, increasing protein adsorption by mineralized matrix deposition ^7^ ^,^ ^8^ ^,^ ^9^, ^10^ ^)_^. The effects of this modification have been elucidated for approximately 10 years, and several alkalinization protocols have been evaluated and standardized with respect to composition and exposure time. Currently, alkalinization by thermal immersion in a sodium hydroxide (NaOH) solution is a promising alternative ^10^ ^).^

In addition to accelerating the osseointegration process, titanium surface modifications can act on the peri-implant tissue response in the face of peri-implant inflammation. The peri-implant disease is characterized by the presence of inflammatory reactions that affect the peri-implant tissues under function, that is, after receiving the implant or much-implant-supported prosthesis. Clinical signs vary from an inflammation restricted to the peri-implant mucosa, and mucositis to bleeding on probing, suppuration, clinical attachment loss, and bowl-shaped bone seen on radiographs ^11^ ^,^ ^12^ ^)_^.

Microbiological evaluations of peri-implant diseases have demonstrated the presence of different pathogens, organized in dual or even multi-species biofilms ^13^ ^)_^ *.* Dysbiosis among the microbiota, its products, and host results in increased expression of inflammatory mediators, which act as potent inducers of the inflammatory response and may be directly or indirectly related to bone metabolism and remodeling ^14^ ^)_^.

Thus, modulation of the tissue response through surface modifications, creating bioactive surfaces, could result in acceleration of the peri-implant maintenance process and also help maintain the homeostasis of peri-implant tissues, as well as improve responsiveness to inflammatory/infectious stimuli. Therefore, this study evaluated human osteoblast behavior, using an *in vitro* implant model, cultured on polished titanium surfaces or subjected to two surface modification protocols by alkaline and heat treatment with sodium hydroxide (NaOH).

## Material and methods

### Obtaining, standardizing, and characterizing titanium disks

Titanium disks of 2-mm thickness were obtained by machining from titanium grade 2 sampling cylinders; 25-cm long and 13 mm in diameter (Realum Industry and Trade of Pure Metals and Alloys, São Paulo, SP, Brazil).

The disks were manually polished using water-based sandpapers with 400, 600, and 1200 grit sizes (T469-SF- Norton, Saint-Gobam Abrasivos Ltda., Jundiaí, SP, Brazil). To eliminate titanium fragments and surface organic material, the disks were cleaned in solutions of acetone P.A., deionized water, 100% ethanol, and deionized water for 15 min each, in an ultrasonic bath.

### Surface Modifications

Different titanium surfaces were evaluated; the polished surface, considered as the control group, which was obtained from the initial polishing of the titanium disks, as previously described; and the surfaces modified with alkaline and heat treatment, along with NaOH at two different temperatures, 60°C and 120°C, as described below:

A 5 M (5 mol/L) NaOH solution (Sigma-Aldrich, St Louis, MO, USA) was prepared in deionized water. After initial polishing, an autoclave was used for cleaning and sterilization, and the titanium disks were immersed in this solution and incubated for 24 h at 60°C or 120°C, under agitation. After hydrothermal treatment, the disks were washed in sterile deionized water for 15 min.

### Evaluation of the roughness and topography of titanium disks

The topography and surface roughness of polished titanium disks or those subjected to surface modification by alkaline and heat treatment with NaOH at different temperatures were determined using scanning electron microscopy (SEM), associated with analysis in ImageJ software. For this, the disks were subjected to gold metallization and evaluated under a scanning electron microscope (Inspect Scanning Electron Microscope-S50; FEI, Hillsboro, OR, USA) at 10,000X magnification. The surface topography was determined using qualitative analysis. For the quantitative analysis of surface roughness, five equidistant linear regions of each sample (replicates) were evaluated, whose average value was considered as the disk surface roughness value (sample unit, n = 6).

### Cell culture

This study was performed using an *in vitro* implant model in which cells were previously cultured on titanium disks to simulate the contact of osteoblasts with oral implants. This model allows greater mimicry of the cell's response in direct contact with the materials/surfaces to be evaluated.

For this study, the SaOs-2 human osteoblast lineage (American Cell Culture-ATCC# HTB85) was used. The cells were maintained in the Dulbecco’s Eagle Culture Medium (DMEM) (Gibco, Carlsbad, CA, USA), supplemented with 1% antibiotic solution (Penstrep, Gibco) and 10% fetal bovine serum (FBS) (Gibco). After thawing, the cells were incubated at 5% CO_2_ and 37°C. Subcultivation procedures were performed every 72 hours using trypsin/EDTA 0.25% (Gibco). To perform the experimental protocols, after allocating the disks into 24-compartment plates, 1 mL of DMEM containing 1% antibiotic solution and 10% FBS was added to each compartment of the plate. Subsequently, the cells were disaggregated (trypsin/EDTA 0.25%, Gibco), counted, and transferred to the disk surfaces at a density of × 10^4^ cells per disk, followed by incubation at 37°C and 5% CO_2_ for different periods, according to the proposed analysis protocols.

### Cell morphology analysis

The morphology of osteoblasts adhered to different titanium surfaces was analyzed using SEM (n = 2). After 24 and 48 h of cultivation on the different surfaces, the samples were fixed in 2.5% glutaraldehyde for 1 h, followed by post-fixation with 1% osmium tetroxide for 1 h. The samples were then washed in deionized water and subjected to dehydration with increasing concentrations of ethanol (30%, 50%, 70%, 95%, and 100%), for 30 min each and finally, subjected to chemical drying with 1,1,1,3,3,3 Hexamethyldisilazane (HMDS) (Sigma-Aldrich), for 20 min, for three repetitions. After these procedures, the titanium disks were metalized with gold and analyzed under a scanning electron microscope (Inspect Scanning Electron Microscope-S50, FEI, Hillsboro, OR, USA) at 1000X and 5000X magnification.

### Analysis of osteoblast adhesion to substrates

The adhesion of osteoblasts to titanium surfaces was determined by direct fluorescence at 24 and 48 h after culture using a cytoskeleton marker (Actin Red, Invitrogen 650 nm) and a DNA intercalator (Hoescht, Invitrogen) (n = 6). In each proposed period, cells were fixed in 4% paraformaldehyde for 30 min, washed in 1X PBS, permeabilized with Triton x-100 (0.1%) (Sigma-Aldrich), and incubated with Actin Red for 30 min. Subsequently, the cells were washed again in phosphate buffer and nuclear stained with Hoescht (1:5000) for 15 min. The samples were qualitatively analyzed under fluorescence microscopy (Leica DM6000, Leica Microsystems, Wetzlar, GE). Four fields from each sample were photographed and quantitatively analyzed in ImageJ software.

### Cell viability analysis

The viability of osteoblasts was determined using the PrestoBlue assay (Invitrogen, Carlsbad, CA, USA). This assay is based on the oxireduction of resazurin to resorufin, which results in the emission of fluorescence whose intensity is directly proportional to the number of viable cells. Thus, PrestoBlue solution was added to the cells at a concentration of 10% in FBS-free DMEM. After 10 min of incubation at 37°C, the fluorescence intensity (560 or 590 nm) (Synergy H1 Hybrid Multi-mode Microplate Reader-Biotek, Winooski, VT, USA) was determined. The average of the results obtained for the polished disk group was considered as 100% viability and all groups were evaluated in comparison to this one.

### Collagen Synthesis Evaluation

Collagen synthesis was determined using the Sirius Red assay. For this analysis, the culture medium in contact with the cells was collected and stored at −20°C until the moment of testing. 200 μL of the culture medium was used and added to a tube containing 200 μL of 0.1% Direct Red reagent (Sigma-Aldrich, St Louis, MO, USA). This solution was then incubated under stirring for 1 h (400 rpm) (Thermomixer, Eppendorf, Hamburg, Germany) at room temperature (25°C). After incubation, the samples were centrifuged at 10^4^ rpm for 10 min (Microcentrifugal 5415R, Eppendorf, Hamburg, Germany), and a pellet was obtained, which was washed in hydrochloric acid solution (0.1 mol/L) and centrifuged again with the same parameters. Finally, the pellet was solubilized in sodium hydroxide solution (0.5 mol/L). Three 100 μL aliquots of each compartment were transferred to a 96-compartment plate (Costar Corp., Cambridge, MA, USA) and evaluated using a spectrophotometer at 555 nm (Synergy H1).

### Total protein synthesis analysis

To determine total protein production by osteoblasts cultured on the different surfaces, cells were lysed in 1 mL 0.1% sodium lauryl sulfate at room temperature (25°C) for 40 min. Subsequently, 1 mL of Lowry’s solution was added, followed by incubation for 20 min. Finally, 0.5 mL of Folin & Ciocalteu’s Phenol reagent, previously diluted in deionized water (1:5), was added. After incubation for 30 min in the absence of light, three aliquots of 100 μL from each compartment were transferred to a 96-compartment plate (Costar Corp.) and the samples were subjected to evaluation in a spectrophotometer at 655 nm (Synergy). The total protein concentration was determined from a standard curve containing previously established concentrations of bovine albumin (Sigma-Aldrich).

### Evaluation of Alkaline phosphatase activity

Alkaline phosphatase (ALP) activity was determined following an endpoint assay (Labtest Diagnóstico S.A., Lagoa Santa, Minas Gerais, Brazil). The reaction was prepared according to the manufacturer’s recommendations, using 50 µL of each sample, followed by incubation at 37°C for 10 min. After this period, 2 mL of colorimetric reagent (sodium carbonate 94 mmol/L and sodium hydroxide 250 mmol/L) was added. After checking the homogeneity of the solutions, three 100 μL aliquots of each sample were transferred to a 96-compartment plate (Costar Corp.). The samples were analyzed in a spectrophotometer (Synergy H1) at a wavelength of 590 nm. The ALP activity was calculated using a standard curve with predetermined values of the enzyme, established from the standard reagent provided in the kit. The ALP activity results were normalized by the total protein production of each sample.

### Gene expression analysis

To evaluate gene expression, the cells were cultured on the disks in complete DMEM and maintained for 24 h. They were exposed to inflammatory stimulation with LPS from *Porphyromonas gingivalis* (*P.gingivalis*) (1 µg/mL) in FBS-free medium for 4 h. After this period, samples were collected to evaluate the expression of tumor necrosis factor-alpha (TNF-α) and beta-defensin 3 (HBD3).

Isolation of total RNA was performed using the RNAqueous™ Kit (Applied Biosystems, Carlsbad, CA, USA) using a filtration system. Next, the RNA concentration of each sample was determined in a spectrophotometer (Take3 System - Synergy H1). For each RNA sample obtained, cDNA (complementary DNA) was synthesized and samples were subjected to the amplification cycle recommended by the manufacturer (Applied Biosystems): 25°C (10 min), 37°C (120 min), 85°C (5 s), 4°C (∞).

After the cDNA synthesis, the expression of genes encoding TNF-α and HBD3 was evaluated by quantitative PCR (polymerase chain reaction). Reactions were prepared with standardized real-time PCR reagents, Syber Green, Universal PCR Master Mix (Applied Biosystems), adding the gene-specific primer sets. Fluorescence readings were taken at each amplification cycle, using the Step One Plus (Applied Biosystems) and later analyzed by Step One Software version 2.1 (Applied Biosystems). All reactions were normalized by the ROX passive reference dye signal to correct for fluctuations in the reading due to volume variations and evaporation throughout the reaction, and the result, expressed as CT value (referring to the number of PCR cycles required for the fluorescent signal to reach the detection threshold) were normalized according to the expression of the selected endogenous gene (RPL13). Data concerning the primers used are presented in Table 1.

**Table 1:** Primers used for gene expression analysis.

Target Gene	Primers
HBD3	Forward: 5'-GCTATGAGGATCCATTATCTTCTG-3' Reverse: 3'-TTATTTCTTTCTTCGGCAGCATTTTC-5'
TNF-α	Forward: 5'-CCCAGGCAGTCAGATCATCTTC-3' Reverse: 5'-AGCTGCCCCTCAGCTTGA-3'
RPL13	Forward: 5'-CCGCTCTGGACCGTCTCAA-3' Reverse: 5'-CCTGGTACTTCCAGCCAACCT-3'

### Data analysis

Data regarding surface topography and cell adhesion were evaluated qualitatively and presented descriptively. The quantitative data regarding the evaluations of surface roughness, cell viability, collagen synthesis, total protein production, and ALP activity were subjected to distribution and homogeneity analysis (Levene test) and further analyzed by one-way ANOVA and Tukey’s statistical tests. Data concerning the evaluation of gene expression were analyzed using the two-way ANOVA test, followed by Tukey’s post-test.

## Results

### Topography and surface roughness

**Figure 1:**
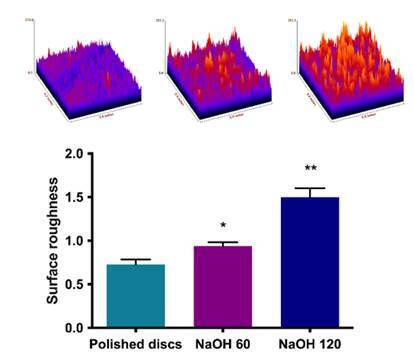
Topography of the surfaces of polished disks and subjected to alkaline treatment at 60°C or 120°C. Surface roughness of polished titanium disks and subjected to alkaline treatment at 60°C or 120°C. Bars indicate mean and standard deviation. Groups identified with different symbols are statistically different from each other (Tukey, p < 0.05).

The analysis of the surface topography of the polished disks subjected to alkaline hydrothermal treatment with NaOH at different temperatures showed that greater irregularities (peaks and valleys) were observed for the treated groups, especially for the disks subjected to immersion at 120°C (Figure 1). The quantitative analysis of surface roughness corroborated this analysis so that the titanium disks subjected to treatment in NaOH solution at 120°C showed the highest roughness values, followed by the disks treated at 60°C and the polished disks (p < 0.01) (Figure 1).

### Cell morphology

**Figure 2:**
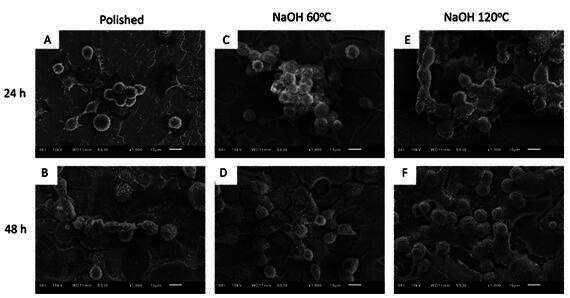
Morphology of osteoblasts adhered to the surfaces of polished titanium disks and subjected to alkaline treatment at 60°C or 120°C, using SEM, after 24 and 48 h of cell culture. The white arrow demonstrates rounded cells with weak adhesion to the substrate, while the blue arrow demonstrates cells with high spreading over the disk.

The analysis of cell morphology by means of SEM showed a larger number of cells adhered to the surfaces treated with NaOH solution, with no difference between them. By 48 h, the high spreading of osteoblasts over these surfaces can also be seen (Figure 2).

### Cell adhesion

**Figure 3:**
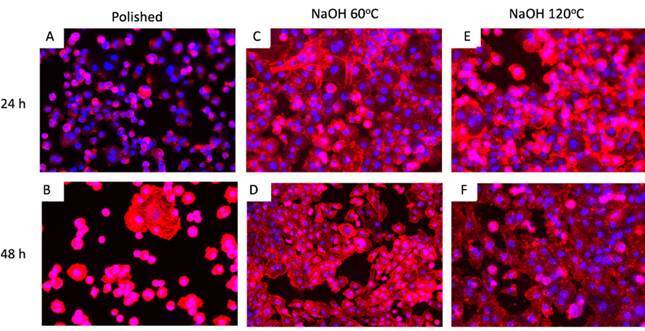
Morphology of osteoblasts adhered to the surfaces of polished titanium disks (a,b) and subjected to alkaline treatment at 60°C (c,d) or 120°C (e,f), by fluorescence microscopy, after 24 and 48 h of cell culture. In blue, you can identify the nucleus of the adhered cells, while the actin filaments of the cytoskeleton are stained in red.

Cell adhesion analysis by fluorescence microscopy showed a high number of cells adhered to titanium surfaces subjected to surface modification for both periods, with no difference between the protocols. In the 48 h period, a greater spreading of cells was observed on such surfaces than on the polished titanium surface, as evidenced by the marking of actin filaments of the cell cytoskeleton (in red) (Figure 3).

### Cell viability

**Figure 4:**
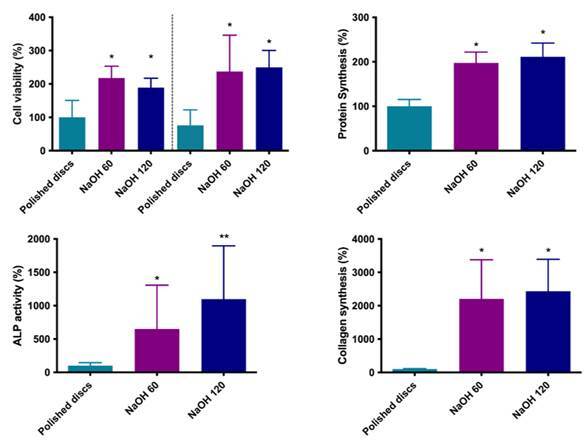
Viability of osteoblasts cultured on polished titanium disks and subjected to alkaline treatment at 60°C or 120°C, after 24 and 48 h. Bars indicate mean and standard deviation. Groups identified with symbols indicate statistically significant differences (n = 6; Tukey, p < 0.05). Collagen synthesis by osteoblasts cultured on polished titanium disks and subjected to alkaline treatment at 60°C or 120°C after 7 days. Bars indicate mean and standard deviation. Groups identified with different symbols indicate a statistically significant difference (n = 6; Turkey, p < 0.05). Total protein synthesis by osteoblasts cultured on polished titanium disks and subjected to alkaline treatment at 60°C or 120°C after 7 days. Bars indicate mean and standard deviation. Groups identified with different symbols indicate a statistically significant difference (p < 0.05). ALP activity by osteoblasts cultured on polished titanium disks and subjected to alkaline treatment at 60°C or 120°C, after 7 days. Bars indicate mean and standard deviation. Groups identified with different symbols indicate a statistically significant difference (p < 0.05).

Osteoblasts cultured on the treated surfaces showed higher cell viability than those cultured on the polished titanium disks (p=0.03), for both periods of analysis (24 and 48 h), with no difference between the treatment protocols (Figure 4).

### Collagen synthesis

Collagen synthesis was significantly higher for osteoblasts cultured on titanium surfaces treated with NaOH for 24 h at 60°C or 120°C, with no difference between protocols (p<0.01) (Figure 4).

### Cell morphology

The analysis of cell morphology by means of SEM showed a larger number of cells adhered to the surfaces treated with NaOH solution, with no difference between them. By 48 h, the high spreading of osteoblasts over these surfaces can also be seen (Figure 2).

### Cell adhesion

Cell adhesion analysis by fluorescence microscopy showed a high number of cells adhered to titanium surfaces subjected to surface modification for both periods, with no difference between the protocols. In the 48 h period, a greater spreading of cells was observed on such surfaces than on the polished titanium surface, as evidenced by the marking of actin filaments of the cell cytoskeleton (in red) (Figure 3).

### Cell viability

Osteoblasts cultured on the treated surfaces showed higher cell viability than those cultured on the polished titanium disks (p=0.03), for both periods of analysis (24 and 48 h), with no difference between the treatment protocols (Figure 4).

### Collagen synthesis

Collagen synthesis was significantly higher for osteoblasts cultured on titanium surfaces treated with NaOH for 24 h at 60°C or 120°C, with no difference between protocols (p<0.01) (Figure 4).

### Total protein production

Total protein production was similar for the experimental groups in which osteoblasts were cultured on the treated surfaces, with no difference between the protocols. Such production was significantly higher than compared to the polished disk group (p=0.03) (Figure 4).

### ALP activity

ALP activity was significantly high for osteoblasts cultured on the treated surfaces, with 120°C treatment resulting in the highest activity values, followed by 60°C protocol (p<0.01)(Figure 4).

### Beta-defensin and TNF-alpha gene expression

**Figure 5:**
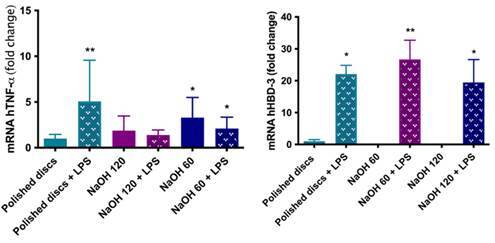
Gene expression of TNF-α and HBD-3 by osteoblasts cultured on polished titanium disks and subjected to alkaline treatment at 60°C or 120°C after 7 days. Bars indicate mean and standard deviation. Groups identified with different symbols indicate a statistically significant difference (p < 0.05).

The analysis of osteoblasts' response to LPS exposure when cultured on different surfaces showed an increase in TNF-α expression by these cells when cultured on the polished disks (p=0.04). For the NaOH-treated disks, there was no difference between the groups exposed or unexposed to LPS. Regarding the expression of HBD3, all groups showed an increase in this expression in the presence of LPS (p<0.05), with this expression being more evident for the group of disks subjected to NaOH treatment at 60°C (Figure 5).

## Discussion

The adhesion of osteoblasts to implants and, consequently, the osseointegration process can be improved by increasing the surface roughness of these implants ^15^ ^,^ ^16^ ^)_^. Several modification methods can be applied to obtain a rough surface; however, alkalinization has shown great promise because it associates this effect with others, such as the antimicrobial effect and the formation of a surface layer that facilitates the carriage of organic molecules ^7^ ^)_^.

This study evaluated the effect of alkalinizing titanium surfaces with sodium hydroxide on human osteoblasts using an *in vitro* model, which simulates the interaction of bone tissue cells with implant surfaces by directly culturing these cells on previously standardized titanium disks according to each experimental group ^17^ ^)_^.

Prior to the analyses on osteoblast behavior, the samples were subjected to morphological characterization. The results demonstrated an increase in surface topography and roughness from both alkalinization protocols; however, more evident for high temperatures (120°C), as previously demonstrated ^18^ ^)_^.

The analysis of the surface topography of the disks subjected to alkaline and heat treatment with NaOH at different temperatures showed that greater irregularities (peaks and valleys) were observed compared to the polished ones, especially for the disks immersed at 120°C. The quantitative analysis of surface roughness corroborated with this analysis, so that the titanium disks subjected to treatment in NaOH solution at 120°C presented the highest values of roughness, followed by the disks treated at 60°C and polished disks (p < 0.05), which may facilitate the rapid bone accumulation and, therefore, promote protein adsorption, cell adhesion, and proliferation, gene expression of inflammatory mediators by osteoblasts cultivated on the different surfaces and exposed to inflammatory stimulus, achieving better tissue integration.

As for osteoblast adhesion, both alkalinization protocols were positive in inducing cell adhesion to titanium surfaces, which showed a greater number of adhered cells, as well as greater spreading of these cells when compared to polished disks. This effect can be justified both by the increased roughness, which has been shown to improve the interaction of mesenchymal cells, and also by the biomodification caused by the alkalinization process, which facilitates the attraction and interaction of organic molecules, such as growth factors, also aiding the adhesion of different cell types ^19^.

Similarly, osteoblast metabolism was also positively affected by the alkalinization protocols, demonstrated by increased viability, total protein and collagen synthesis, and ALP activity. Such processes are directly related to the peri-implant maintenance process, which is determined by the adhesion of osteoblastic cells and the deposition of collagen matrix and mineralization of this matrix ^20^.

Overall, osteoblast metabolism was stimulated similarly for both alkalinization protocols. However, ALP activity was significantly high for cells cultured in disks treated with NaOH at 120°C, which must be a result of high topographic irregularity (roughness) ^10^ ^)_^. Thus, this protocol, when applied in isolation, seems to be more promising in accelerating tissue mineralization and may also be more advantageous from a mechanical point of view.

Modifying the implant surface at the micrometer scale can improve the contact between bone and implant and thus achieve biomechanical improvements and compatibility, which provides an enabling environment for contact osteogenesis access and signaling for tissue-implant interactions. Surface modification and the presence of nanoscale features on the implant surface can stimulate osteoblastic cell proliferation. The reason behind this is the similarity of nanometer features to the biological environment of osteoblasts ^21^.

Currently, it is known that the physical and chemical characteristics of the implant surface are essential factors affecting the rate and extent of osseointegration ^21^ ^)_^. In terms of surface physical specifics, recent studies have highlighted that nanostructured titanium may have better osseointegration than titanium with microscale surface modifications because nanoscale surfaces have a large surface area and can better simulate the extracellular matrix to facilitate rapid bone accumulation and therefore promote protein adsorption, cell adhesion and proliferation, gene regulation, and tissue integration ^21^.

Despite the large amount of data in the literature demonstrating the positive effects of inducing increased surface roughness of implants in the osseointegration process, several protocols can be applied to achieve this change in topography and roughness ^6^ ^)_^. The subtraction treatments by acid etching were precursors in the modification of titanium surfaces ^6^ ^)_^, which resulted in obtaining surfaces with high porosity; however, these treatments also require the use of strong acids, generating exothermic reactions, and can generate residual compounds ^22^ ^)_^. In contrast, the modification of titanium surfaces by alkalization with NaOH is shown to be more effective and promising, as it does not result in residues and risks, as well as facilitates the carriage of molecules that could accelerate the peri-implant maintenance process ^19^ ^,^ ^21^.

Regarding the responsiveness to LPS stimulation, cells cultured on alkalinized disks showed less TNF-α synthesis when compared to those seeded on polished disks. This result, analyzed in isolation, can be interpreted in two ways: At first sight, it can favor tissue repair, reducing the activation of proteolytic enzymes, and accelerating the proliferative phase; or it can reduce the response capacity against bacterial colonization. However, in the present study, gene expression of the HBD3, which acts in the innate immune response to different pathogens, was also evaluated ^23^ ^)_^, demonstrating that cells cultured on the alkalinized surfaces showed greater activation of HBD3 expression in the presence of LPS of *P. gingivalis.*

The development and optimization of treatment surfaces for implants are crucial for the acceleration of bone repair, mainly for patients who may present delayed healing, such as diabetic and osteoporotic patients. Moreover, the elucidation of the effect of these materials on bone cells is also very relevant to determining whether these new strategies may be clinically suitable. This investigation confirmed the promising effects of alkalinization on osteoblasts' behavior. However, despite the positive results observed in the present study, we must consider the limitations that it is a cell-culture study, and therefore results should be considered with caution, considering the design applied in the study.

## Conclusions

Titanium surface modifications with NaOH at 60 and 120^o^C improve osteoblast adhesion and response to inflammatory stimulus.
